# The geographical distribution of grey wolves (*Canis lupus*) in China: a systematic review

**DOI:** 10.13918/j.issn.2095-8137.2016.6.315

**Published:** 2016-11-18

**Authors:** Lu WANG, Ya-Ping MA, Qi-Jun ZHOU, Ya-Ping ZHANG, Peter SAVOLAINEN, Guo-Dong WANG

**Affiliations:** ^1^State Key Laboratory for Conservation and Utilization of Bio-resources in Yunnan, Key Laboratory for Animal Genetic Diversity and Evolution of High Education in Yunnan Province, Yunnan University, Kunming 650091, China; ^2^State Key Laboratory of Genetic Resources and Evolution, Yunnan Key Laboratory of Molecular Biology of Domestic Animals, Kunming Institute of Zoology, Chinese Academy of Sciences, Kunming 650223, China; ^3^Science for Life Laboratory, Department of Gene Technology, KTH-Royal Institute of Technology, Solna 17165, Sweden

**Keywords:** China, Grey wolf, Distribution, Conservation

## Abstract

The grey wolf (*Canis lupus*) is one of the most widely distributed terrestrial mammals, and its distribution and ecology in Europe and North America are largely well described. However, the distribution of grey wolf in southern China is still highly controversial. Several well-known western literatures stated that there are no grey wolves in southern China, while the presence of grey wolf across China has been indicated in *A Guide to the Mammals of China*, published by Princeton University Press. It is essential to solve this discrepancy since dogs may have originated from grey wolfs in southern China. Therefore, we systematically investigated Chinese literatures about wild animal surveys and identified more than 100 articles and books that included information of the distribution of grey wolves in China. We also surveyed the collections of three Chinese natural museums and found 26 grey wolf skins specimens collected across China. Moreover, we investigated the fossil records of wolf in China and identified 25 archaeological sites with wolf remains including south China. In conclusion, with the comprehensive summary of Chinese literatures, museum specimens and fossil records, we demonstrate that grey wolves does distribute across all parts of the Chinese mainland, including the most southern parts of China.

## INTRODUCTION

The grey wolf, *Canis lupus*, is one of the most widely distributed terrestrial mammals (Young & Goldman, 1944). Grey wolves live in a wide variety of habitats, including the dry Arabian desert, the xeric Mediterranean shrublands, the coniferous forests of Siberia, and the frozen tundra on Ellesmere island (Mech, 1981). Despite extirpation from many parts of their previous range over the last few hundred years, by persecution from humans and habitat fragmentation (Hunter & Barrett, 2011; Young & Goldman, 1944), wolves still retain most of their original distributions.

The distribution and ecology of grey wolves are largely well described in Europe and North America. However, in more peripheral and remote parts of its distributions, detailed information is often lacking. In the western literature, the wolf has generally been reported to be distributed throughout the northern hemisphere, from N15° latitude in North America and N12° latitude in India to beyond the Arctic Circle, but has been considered to be absent from Africa and the southern East Asia (Mech, 1981). However, recent articles reported that the Egyptian jackal (*Canis aureus lupaster*, Hemprich and Ehrenberg 1833) is not a subspecies of the golden jackal (*Canis aureus*, Linneaus 1758) and should be reclassified as the African wolf, *Canis lupus lupaster* (Gaubert et al., 2012; Koepfli et al., 2015; Rueness et al., 2001).

Similarly, the literature about wolves in China is limited outside China. This has led to misconceptions in the western literature about the distributions of wolves in China. Four studies, all conducted by western researchers, stated that wolf has never been presented in large parts of China (Callaway, 2013; Larson & Fuller, 2014; Nowak, 2003; Sokolov & Rossolimo, 1985). 

However, as will be shown in this report, the wolf has a historical and current range across nearly the entire country of China. There are more than 100 Chinese articles and books involving investigations of wolves in China since the 1950s (Table 1), showing the distributions in detail. Most of these articles are species investigations at a provincial or local level, however, and there is no comprehensive description of the current distribution of wolves across China. Therefore, we here summarize the Chinese literature concerning past and present distributions of wolves in China, in order to synthesize data from this rich source of regional investigations into a comprehensive map of wolf distribution in China, and to make this significant information available to an international audience.

**Table 1:** Literature list of distributions of wolves in China

Province	Investigation Year	Location	Reference
Heilongjiang	2008-2009	Eastern forests of Wandashan Mountains	Shen et al., 2011
	1994-2001	Sanjiang National Reserve	Zhang et al., 2001
	1997-1999	Tangwanghe river forest distict	He et al., 2003
	1993-1995	Grand Khingan	Zhang et al., 1998a
	1988-1989, 1993-1995	Northern Grand Khingan	Li et al., 1996
	1984, 1987-1990, 1992	Xingkai Lake Nature Reserve	Li et al., 1993
	1971-1980, 1981-1990	Heilongjiang province	Zhang et al., 1998b
	N/A	Heilongjiang province	Zhang & Yu, 2005
	N/A	Western Helongjiang province	Gao et al., 1999
Jilin	1992-1993	Yanbian	Qiu et al., 1995
	1992-1993	Hunjiang	Li et al., 1994
Liaoning	1999-2002	Nuluerhusan National Reserve	Zhou et al., 2007
	1996-2000	Benxi	Zhao et al., 2004a
	1996-2000	37 counties in Liaoning province	Zhao et al., 2004b
	1996-1999	Fushun	Zhao et al., 2001
	N/A	Yiwulv Mountain National Nature Reserve	Liu et al., 2008
	N/A	Liaoyang	Wang et al., 2004
Inner Mongolia	1985-1986	Jiufeng Shan	Liu & Liu, 1999
	N/A	Chaihe	Xiao et al., 2013
	N/A	Hulunbair & Hinggan	Gao et al., 1999
Beijing	1982-1983	Changping & Miyun	Zhang, 1984
	N/A	Beijing	Wu et al., 2006
Tianjin	N/A	Tianjin	Wu et al., 2006
Shanxi	2010-2011	Pangquangou National Nature Reserve	Wang & Zhao, 2011
	1996-1997	Luyashan Nature Reserve	Qiu et al., 1998
	N/A	Northeastern Loess Plateau	Chen, 2000
Hebei	1993-2001	Chengde	Hou et al., 2004
	N/A	Hebei province	Wu et al., 2006
	N/A	Saihanba	Hou et al., 1994
Gansu	2007-2009	Sunan and Subei prairie	Zhao et al., 2011
	N/A	Gannan plateau	Chen & Li, 1994
	N/A	Longnan mountain	Chen et al., 1994
	N/A	Tianshui	Hu et al., 1993
	N/A	Minqin desert	Chen, 1992
	N/A	Anxi	Chen & Luo, 1991
Xinjiang	1994-1996	Kanas National Nature Reserve	Abdukadi et al., 1999
	1987-1988	Wuqia, Taxkorgan, Yecheng, Qiemo, Yutian	Feng, 1990
	1965, 1980, 1983, 1985	Zhungeer & Altai	Zhang & Hu, 1988
	1979	Xinjiang	Gao, 1997b
	1958-1961	Desert plains area in Xinjiang	Zhang, 1963
	N/A	West Tianshan National Nature Reserve	Liu et al., 2007a
Ningxia	2010-2011	Luoshan National Nature Reserve	Qin & Chang, 2012
Shaanxi	2006	Huanglongshan Nature Reserve	Li & Liu, 2009
	2006	Micangshan Nature Reserve	Wen et al., 2008
	1997-2000	Changqing National Nature Reserve	He, 2001
	1999	Zhashui county	Hu et al., 2003
	1996	Zhouzhi National Nature Reserve	Li & He, 1997
	1963-1966	Ankang	Wu & Li, 1982
	1959	Daba mountain	Wang et al., 1981
	N/A	Shaanxi province	Li et al., 2006
Qinghai	2001-2002	Qilian mountain	Xia et al., 2003
	N/A	Beichuan River Nature Reserve	Zhang & Pu, 2012
	N/A	Qinghai lake area	Kong et al., 2011
Tibet	2001-2002	Upper Zayu river basin	Wu, 2006
	1987-1988	Ngari & Naqu	Feng, 1990
Sichuan	2006	Kasha Lake Nature Reserve	Liu et al., 2013
	1997, 2006	Ruoergai Wetland National Nature Reserve	Liu et al., 2009
	2005-2006	Maozhai Nature Reserve	Liu et al., 2007b
	2003-2005	Haizishan Nature Reserve	Liu et al., 2007c
	2004	Heizhugou Nature Reserve	Liu et al., 2005a
	2002-2003	Jiuzhaigou National Nature Reserve	Liu et al., 2005b
	2002-2003	Dafengding Nature Reserve	Liu et al., 2004
	2002-2003	Yele Nature Reserve	Zhang & Hu, 2004
	2001-2002	Huanglong Nature Reserve	Zhu et al., 2010
	2002	Xuebaoding Nature Reserve	Sun et al., 2006
	2001	Pingwu	He et al., 2004
	1998	Big-small Langou Nature Reserve	Lu & Hu, 2003
	1996	Huanglongsi Nature Reserve	Hu et al., 2001
	N/A	Ganzi and Liangshan	Zhang et al., 2009
	N/A	Ruoergai Wetland National Nature Reserve	Hao et al., 2008
	N/A	Wolong Nature Reserve	Yu et al., 1983
Yunnan	2010-2011	Lanping Yunling Provincial Nature Reserve	Cui et al., 2014
	2010-2011	Weixi	Zha et al., 2014
	N/A	Yunnan province	Yang et al., 1999
Guizhou	2005-2006	Leigong Mountain National Nature Reserve	Chen et al., 2008
	N/A	Guizhou province	Luo & Li, 2001
	N/A	Weining	Huang, 1989
Chongqing	2006-2008	Jinfo Mountain Natural Reserve	Zong et al., 2010
	1995	Jinfo Mountain Natural Reserve	Peng et al., 1996
	N/A	Chongqing	Han & Hu, 2002
Henan	1997	Xin'an, Yuzhou, Jiyuan, Luoning, Jiaozuo, Zhenping	Gan & Fan, 2004
Hubei	2004	Yerengu Nature Reserve	Wang et al., 2007
	2004	Wudaoxia Nature Reserve	Wu et al., 2005
	2001	Qizimei Mountain Nature Reserve	Liu et al., 2002
	N/A	Duheyuan Provincal Nature Reserve	Li et al., 2008
Hunan	1980-1981	Ziyunshan	Fu, 1987
Jiangxi	2004-2007	Taohong Ridge Sika Deer Nature Reserve	Wu et al., 2012
	1984-1986	Poyang lake area	Fu & Ding, 1991
	N/A	Jiangxi province	Tu et al., 2014
	N/A	Lushan Nature Reserve	Li et al., 2007
Shandong	1984-1987	Jiaodong peninsula	Sun, 1988
	1982-1986	Qingzhou	Cong, 1988
	1961-1966, 1973-1984	Jiaodong and Luzhongnan area	Lu, 1984
	N/A	Laoshan	Tian et al., 2000
Anhui	1959-1964	Anhui province	Wang et al., 1966
	N/A	Anhui province	Wu et al., 2002
	N/A	Huangshan	Xu, 1997
Jiangsu	N/A	Jiangsu province	Wang & Zhao, 2008
Zhejiang	2005-2008	Hangzhou	Ding et al., 2008
	1958-1960, 1962-1964, 1979-1981	Zhejiang province	Zhuge, 1982
	N/A	Jinhua	Zhu & Yu, 1996
	N/A	Yongkang	Bao & Hu, 1987
Fujian	N/A	Fujian province	Chen et al., 2009
	N/A	Fujian province	Zhou, 1997
	N/A	Fujian province	Zhan, 1995
Guangxi	1997-2000	Shiwan Mountain	Xia et al., 2002
	1958	Southwestern Guangxi	Wang et al., 1962
Guangdong	2000	Nanling National Nature Reserve	Fellowes et al., 2003

## LITERATURE SUMMARIZATION

It is controversial to describe the distribution of grey wolves in western literatures. Two articles reported that wolves were previously present all across China, but is now extinct from southern China (Ginsberg & Macdonald, 1990; Lau et al., 2010). In four well-known studies, researchers claimed that wolves have never existed in sourthern China (Callaway, 2013; Larson & Fuller, 2014; Nowak, 2003; Sokolov & Rossolimo, 1985), suggesting that sourthern China cannot be the harbor of dog domication. Thus, southern China is usually treated outside the range of wolf distribution (IUCN; EO). However, in 2008, Smith and his colleagues described the distribution of wolf in China, indicating that grey wolves were present all across the mainland of China (Smith & Xie, 2008).

In the Chinese literature, wolves have been reported to appear over all parts of continental China. The Fauna Sinica (China): Mammalia Vol. 8 Carnivora page 46-49, reported in 1987: "the wolf, which apart from Hainan Island, the various islands in the South China Sea, and Taiwan, is spread over nearly all the country" and "the wolf can be seen in all provinces. Based on collected literature references and specimen samples, wolves have been identified in Muleng, Baoqing, and Genhe of Heilongjiang, in Baicheng, Kaitong, Dunhua, Jingyu, Huinan, Hunchun, Jilin, Tumenling, and Fuyu of Jilin, in Fushun and Lvda of Liaoning, in Shanhaiguan and Zhangjiakou of Hebei, in Beijing, in Hohhot and Erlian of Inner Mongolia, in Hami, Bole, Turpan, Yanqi, Korla, Aksu, Luntai, and Baicheng of Xinjiang, In Shanxi province, in Yan'an of Shaanxi, in Mianchi and Luoning of Henan, in Yichang of Hubei, in Nanjing and Qingjiang of Jiangsu, in Fujian province, in Longzhou, Ningming, and Shangsi of Guangxi, in Guangdong province, in Guizhou province, in Lushui and Chengkou of Yunnan, in Yumen, Zhangye, and Linxia of Gansu, in Menyuan, Qilian, Alaer, Golmud, and Delingha of Qinghai, in Pali, Nylamu, Tingri, Shigatse, and Naqu of Tibet, and in Shiqu, Ruoergai, Songpan, Leibo, Ebian, Kangding, Wanxian, Yibin, and Mianyang of Sichuan" (Gao & Wang, 1987).

Furthermore, Wang (200) described the subspecies/subtypes of wolf in China and reported that the wolf was distributed across all parts of continental China. Chinese wolves were divided into five subspecies and forms: *Canis lupus desertorum* Bogdanow, 1882 in Xinjiang, *C*. *l*. *filchneri* Matschie, 1907 in Qinghai, Gansu and Tibet, *C*. *l*. *chanco* Gray, 1863 in Heilongjiang, Jilin, Liaoning, Inner Mongolia (eastern part), Hebei, Beijing, Shandong, Henan and Shanxi, *C*. *l*. Nei-Mongol form in Inner Mongolia (western and mid part) and *C*. *l*. South-China form in Anhui, Jiangsu, Zhejiang, Jiangxi, Fujian, Guangdong, Hunan, Guizhou, Yunnan, Hubei and Sichuan 

**Figure 1:**
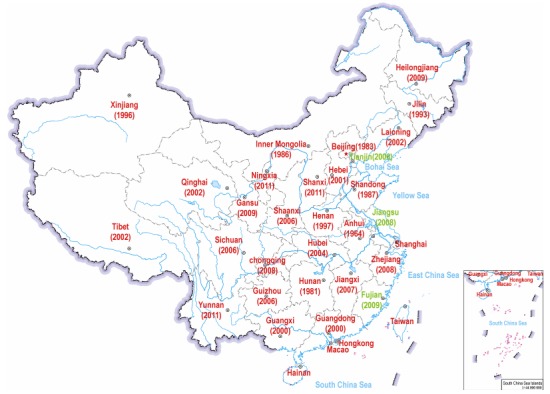
Distributions of wolves in China

In order to obtain an updated and comprehensive description of the distribution of wolves in China, we investigated more than 100 articles containing information about the pressence of wolf at a regional level (see a full list of literature in Table 1). The most recent evidence of wolf in each province (Figure 1) were extracted from the following papers: Heilongjiang (Shen et al., 2011), Jilin (Qiu et al., 1995), Liaoning (Zhou et al., 2007), Inner Mongolia (Liu & Liu, 1999), Beijing (Zhang, 1984), Tianjin (Wu et al., 2006), Shanxi (Wang & Zhao, 2011), Hebei (Hou et al., 2004), Gansu (Zhao et al., 2011), Xinjiang (Abdukadir et al., 1999), Ningxia (Qin & Chang, 2012), Shaanxi (Li & Liu, 2009), Qinghai (Xia et al., 2003), Tibet (Wu, 2006), Sichuan (Liu et al., 2013), Yunnan (Cui et al., 2014), Guizhou (Chen et al., 2008), Chongqing (Han et al., 2010), Henan (Gan & Fan, 2004), Hubei (Wang et al., 2007), Hunan (Fu, 1987), Jiangxi (Wu et al., 2012), Shandong (Sun, 1988), Anhui (Wang et al., 1966), Jiangsu (Wang & Zhao, 2008), Zhejiang (Ding et al., 2008), Fujian (Chen et al., 2009), Guangxi (Xia et al., 2002), Guangdong (Fellowes et al., 2003).

In summary, these investigations showed that the wolf has been recorded in every continental Chinese province between 1964 and the present, except in three provinces (Figure 1 in green). Most notably, wolves were recorded in South China (in Yunnan province) as late as 2011and in the two southernmost provinces (Guangdong and Guangxi) in the year of 2000. From these findings we concluded that wolves are still present across all parts of continental China.

## WOLF SKINS IN ZOOLOGICAL MUSEUMS

**Figure 2:**
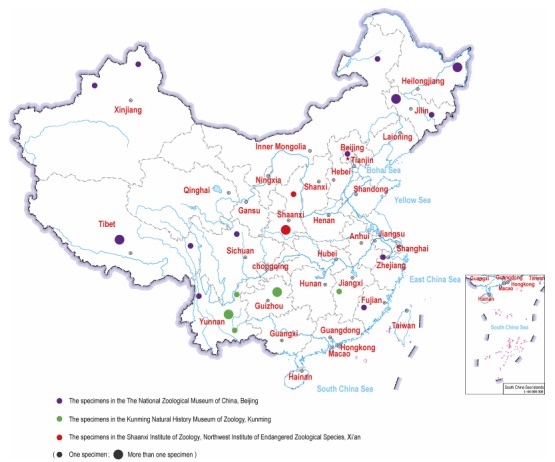
Source and geographical origin of museum wolf skin specimens

**Figure 3:**
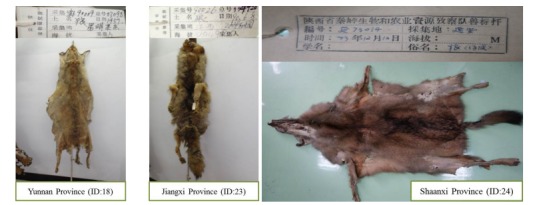
Three museum wolf skin specimens

In addition to the literature investigation, we made a survey of wolf skins in the archives of Shaanxi Institute of Zoology, Kunming Natural History Museum of Zoology and the National Zoological Museum of China (Table 2, Figure 2, Figure 3). We found 26 wolf skins sampled from 13 provinces across China, e.g., two specimens sampled from two southern Chinese provinces (Zhejiang and Fujian) in 1974, and one from southern Yunnan in 1985.

**Table 2:** Sources and geographical origins of wolf skin specimens

Museum	ID	Province	Location	Date
The National Zoological Museum of China, Beijing	1	Heilongjiang	Baoqing	N/A
	2	Heilongjiang	Baoqing	1957.01.24
	3	Inner Mongolia	Xiguitu (Yakeshi)	1954.12.10
	4	Jilin	Baicheng	1957.02.11
	5	Jilin	Jingyu	1956.03.08
	6	Jilin	Kaitong	1956.06.13
	7	Xinjiang	Buerjin	1974
	8	Xinjiang	Bole	1972.05.18
	9	Tibet	N/A	N/A
	10	Tibet	Changdu	1976.1
	11	Tibet	N/A	N/A
	12	Beijing	Yanqing	1984.04.28
	13	Sichuan	Ruo'ergai	1961.07.03
	14	Yunnan	Lushui	1960
	15	Fujian	N/A	1974.05
	16	Zhejiang	Lin'an	1974
Kunming Natural History Museum of Zoology, Kunming	17	Yunnan	Kunming	1967
	18	Yunnan	Kunming	1957
	19	Yunnan	Zhaotong	N/A
	20	Yunnan	Honghe	1985
	21	Guizhou	N/A	N/A
	22	Guizhou	N/A	N/A
	23	Jiangxi	Zoo	1990.06.08
Shaanxi Institute of Zoology, Northwest Institute of Endangered Zoological Species, Xi'an	24	Shaanxi	Yan'an	1973
	25	Shaanxi	Xunyang	1965
	26	Shaanxi	Pingli	1965

## WOLF FOSSIL RECORD

We investigated the literature about archaeological research in China, to identify information about wolf fossils in archaeological sites. We extracted information about the fossil record of the grey wolf in China from three Chinese books (Lv, 2004; Yuan, 2015; Zhang et al., 2003). These books reported 25 archaeological sites in 14 provinces across China with wolf fossils records (Table 3), including the 12 000 years old remains from the South Chinese province Jiangxi.

**Table 3:** Fossil records of gray wolves

Province	County	Archaeological site	Time	Reference	
Shanxi and Hebei	Yanggao and Yangyuan	Xujiayao	About 100 000 years ago	Zhang et al., 2003	p259
Shaanxi	Pucheng	Nanwan and Beiwan	Epipleistocene		p315
Henan	Anyang	Xiaonanhai	22 150-11 000 years ago		p320
Heilongjiang	Harbin	Yanjiagang	22 370±300 years ago		p357
Shanxi and Hebei	Yanggao and Yangyuan	Xujiayao	125 000-104 000 years ago	Lv, 2004	p96
Hebei	Yangyuan	Banjing	108 000-74 000 years ago		p100
Shanxi	Yanggao	Shenquansi	11 720±150 years ago		p102
Liaoning	Haicheng	Xiaogushan	Epipleistocene		p207
Chongqing	Fengjie	Yufupu	7 560±110 years ago		p355
Heilongjiang	Mishan	Xinkailiu	7 500-6 500 years ago	Yuan, 2015	p114
	Qiqihar	Tengjiagang	Bronze age		p115
	Hailin	Xilinhe	Bohai Kingdom (698-926 A.D.)		p115
Jilin	Nong'an	Zuojiashan	6 800-4 800 years ago		p115
Liaoning	Dalian	Guojiacun	5 780-4 300 years ago		P118
Inner Mongolia	Linxi	Baiyingchanghan	8 000-5 000 years ago		P120
	Baotou	Yanjialiang	1 275-1 372 years ago		p127
Shaanxi	Nanzheng	Longgangsi	6 500-6 000 years ago		p130
	Tongchuan	Beicun	Shang Dynasty (1 600-1 046 B.C.)		p133
Hebei	Xushui	Nanzhuangtou	About 10 000 years ago		p144
Beijing	Fangshan	Zhenjiangying and Tazhao	Shang and Zhou Dynasties (1 600-256 B.C.)		p145
Shandong	Yanzhou	Wangyin	6 500-5 500 years ago		p147
	Weifang	Qianbuxia	Houli Culture (8 500-7 500 years ago) and 5 500-5 000 years ago		p147
Tibet	Naqu	Chaxiutang	9th-11th century A.D.		p155
Hubei	Zigui	Liulinxi	Neolithic age, Erlihe Culture (21st-15th century B.C.), and the Eastern Zhou Dynasty (770-256 B.C.)		p158
	Badong	Lijiatuo	Eastern Zhou Dynasty (770-256 B.C.)		p164
Jiangxi	Wannian	Xianrendong	About 12 000 years ago		p166

## DISCUSSION

In this study, we showed that contrary to what is reported in many references in the western literature, the wolf actually is present across virtually all parts of the mainland China. This correction is important in studies of wolf ecology and conservation. It gives a correct picture of the worldwide distributions of wolves, by filling in a large blank region on the map. It is also important in studies of the history of domestic dogs, since dogs probably trace a large proportion of their genetic ancestry to wolves from the southern parts of East Asia (Wang et al., 2016).

The wolf has endured massive decline in population size and geographic range around the world during the previous two centuries, because of human influence including habitat loss, persecution, hunting (for obtaining, e.g., trophies, furs and material for traditional medicine), and depletion of prey (Beschta & Ripple, 2010; Callan et al., 2013; Levi & Wilmers, 2012; Ripple et al., 2014). Also in China, the distribution areas of wolves have severely decreased due to human mediated habitat loss and hunting (Gao, 1997a, 2006; Zhang, 1999). Official investigations from the middle of the 20th century reported that wolves were distributed in every province of China except some islands, but gave no exact numbers. Today, large populations remain only in the northwestern and northeastern parts of the country, Inner Mongolia and Tibet, but even in these regions, the numbers are relatively small, e.g., only 2 000 wolves in Inner Mongolia were reported in the 1990s (Gao, 1997a). We have here shown that wolves still seem to be present across all parts of the Chinese mainland, including the most southern provinces. Thus, even though habitat loss has been severe in urban and agricultural regions, wolves seem to have persisted in intervening regions.

The data about wolf distributions that we here present were investigations on either provincial or local level, whereas, a comprehensive ecological survey of the wolves in China. It is therefore not clear how the wolf populations in the different parts of China are interrelated. For example, it is not clear whether wolves recorded in the southern provinces represent permanent populations, or a steady stream of individuals migrating from the northern provinces. However, it is notable that wolves have been recorded across virtually the entire continental China, including southern Chinese province Yunnan as late as in 2011 and provinces Guangdong and Guangzhou in 2000. These findings indicate a consistent presence of permanent populations across southern China. Moreover, to obtain a comprehensive picture of the status of the wolves in China, it is necessary to carry out both ecological and genetic studies, e.g., in concerning the genetic relationships either among the wolf populations across China and between these and worldwide wolf populations.

This study points out misconceptions in the western literature about the distributions of wolves in China. The origin of this problem is not clear, but it can be traced back as far as an article in 1985 from which the factoid has, stepwise, been passed on to other articles (Sokolov & Rossolimo, 1985). It is probably because of the linguistic barrier to the Chinese literature that this error has previously not been pointed out. This case can be explained by inefficient research in peripheral parts of the species distribution, in countries with limited resources. Our study raises the question whether this kind of misconceptions also exist in other species than just the grey wolf.

## CONCLUSIONS

With a comprehensive summary of Chinese literature, specimens and fossil records, we showed that wolves are present across all parts of the Chinese mainland, including the southern parts of China. Hereby we corrected an error in western literature, in which most sources stated that wolves are not present in the southern China, and some even claimed that wolves have never been presented there, even in ancient times. There is no comprehensive description of the current distributions of wolves across China, and therefore this study serves both to give an updated description of wolf distributions in China, and to make this significant information available to an international audience.
